# Revisiting the Conformational Equilibrium of 1,1,2-Trifluoroethane and 1,1,2,2-Tetrafluoroethane: An NBO Study

**DOI:** 10.1021/acs.jpca.5c06020

**Published:** 2025-10-23

**Authors:** Matheus P. Freitas

**Affiliations:** Department of Chemistry, Institute of Natural Sciences, 67739 Federal University of Lavras, Lavras, MG 37200-900, Brazil

## Abstract

Organofluorine compounds are key to pharmaceutical, agrochemical, and high-performance material applications, where C–F bond conformations influence critical properties such as solubility, lipophilicity, and biological activity. While the conformational behavior of 1,2-difluoroethane and its characteristic *gauche* effect is well understood, the structural preferences of 1,1,2-trifluoroethane and 1,1,2,2-tetrafluoroethane have remained less explored, particularly in light of hyperconjugation theory. In this quantum-chemical study, the conformational equilibria of these two model fluoroalkanes were investigated using density functional theory and Natural Bond Orbital (NBO) analysis, with complementary NMR coupling constant calculations. The results reveal that Lewis-type interactions govern conformational stability, favoring the *anti-gauche* conformer in 1,1,2-trifluoroethane and the *double antigauche* conformer in 1,1,2,2-tetrafluoroethane. Nevertheless, electron delocalization plays an essential role in ensuring that staggered conformations remain energy minima, as fully localized electronic structures would yield only a single conformer for each compound. These findings refine our understanding of hyperconjugative and electrostatic effects in fluorinated ethanes and provide a more nuanced framework for predicting conformational preferences in difluoromethyl-containing systems.

## Graphical abstract

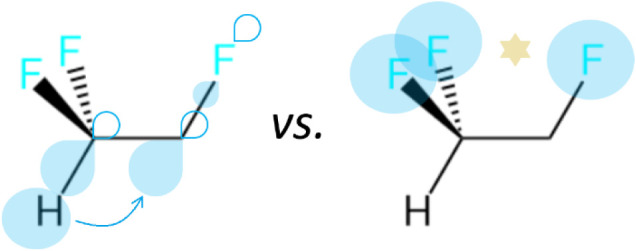

## 1 Introduction

Organofluorine compounds play a significant role in the pharmaceutical, agrochemical, and material industries.^1–8^ In these contexts, the conformation of C–F bonds critically influences properties such as solubility, lipophilicity, and biological activity.^9^ Among fluoroalkanes, 1,2-difluoroethane is the simplest compound that undergoes conformational isomerization and has been extensively studied.^10–14^ Consequently, the factors governing its conformational stability are well established. Notably, 1,2-difluoroethane exhibits the *gauche* effect, a term coined by Saul Wolfe to describe “*a tendency to adopt that structure which has the maximum number of gauche interactions between the adjacent electron pairs and/or polar bonds*.”^15^ This effect arises primarily from antiperiplanar donor–acceptor interactions, specifically σ_CH_ → σ*_CF_ hyperconjugation (Figure 1). It is important to note, however, that Pauli repulsion involving fluorine atoms does not significantly contribute to conformational destabilization in this molecule.^16^ The latest explanation for the *gauche* effect in 1,2-difluoroethane has been proposed by Thacker and Popelier,^17^ who, using the interacting quantum atoms (IQA) approach, attributed the *gauche* stability to 1,3 C···F electrostatic polarization interactions.

**Figure 1:**
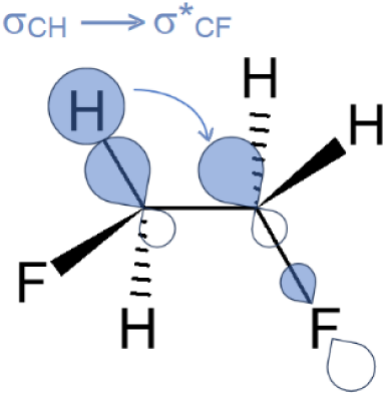
Hyperconjugative interaction responsible for the *gauche* effect in 1,2-difluoroethane.

In contrast, the conformational equilibria of 1,1,2-trifluoroethane and 1,1,2,2-tetrafluoroethane are not as well understood. Most studies on these systems date back to the 1990s,^18–23^ prior to the seminal work of Pophristic and Goodman (2001),^24^ which highlighted the role of hyperconjugation in the rotational barrier of ethane. Earlier investigations provided spectroscopic and theoretical evidence for the conformational preferences of these compounds. For 1,1,2-trifluoroethane, the *anti-gauche* conformer (*gauche* H1–C–C–F2 dihedral angle) has been found to be more stable than the *double gauche* conformer. In contrast, 1,1,2,2-tetrafluoroethane favors the *double antigauche* (nonpolar) conformer (*anti* H1–C–C–H2 dihedral angle) over the *triple gauche* in the gas phase.^18–23^ However, the underlying factors governing these preferences remain poorly understood.

Therefore, this study presents a detailed analysis of the conformational equilibria of 1,1,2-trifluoroethane and 1,1,2,2-tetrafluoroethane (Figure 2) using density functional theory and natural bond orbital analysis. These approaches elucidate the contributions of Lewis-type and non-Lewis-type effects to the overall electronic energy. Lewis-type interactions involve localized, two-center bonds and one-center lone pairs that sterically interact. Although dipolar interactions are not explicitly defined as Lewis-type effects, they are considered under this category here, as electron distributions give rise to charges that repel each other upon close contact. In contrast, non-Lewis-type interactions correspond to delocalization effects arising from two-electron, two-orbital interactions, such as hyperconjugation. In addition, NMR calculations based on spin–spin coupling constants provide complementary insights into the conformer populations. Together, these findings offer an updated perspective on these model organofluorines, deepening our understanding of the conformational behavior of difluoromethyl-containing systems.

**Figure 2:**
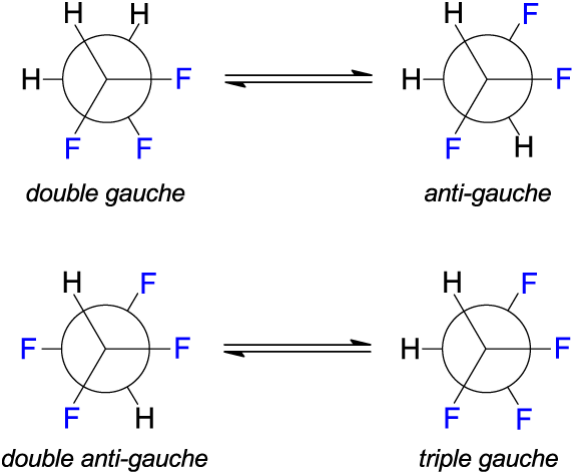
Conformational equilibria of 1,1,2-trifluoroethane and 1,1,2,2-tetrafluoroethane.

## 2 Computational Methods

The H1–C–C–F2 and H–C–C–H dihedral angles of 1,1,2-trifluoroethane and 1,1,2,2-tetrafluoroethane, respectively, were scanned from 0° to 180° in 10° increments at the B3LYP-GD3BJ/6–311++G­(d,p) level.^25–28^ The resulting energy minima were subsequently reoptimized with frequency calculations at the B3LYP-GD3BJ/6–311++G­(d,p)^25–28^ and G3MP2B3.^29^ The optimized geometries were further evaluated through single-point energy calculations at the DLPNO–CCSD­(T)/CBS level.^30,31^ Each point along the potential energy surface (PES), including the optimized minima, was subjected to Natural Bond Orbital (NBO) analysis using the *NBODEL* and *NOSTAR* keywords.^32^ This allowed for the extraction of deletion energies alongside the total electronic energy (E_full_), enabling decomposition into Lewis-type (E_L_) and non-Lewis-type (E_NL_) contributions: E_full_ = E_L_ + E_NL_. The former accounts for steric and dipolar interactions, while the latter captures electron delocalization effects. Additionally, spin–spin coupling constants were computed at each PES point to assess their sensitivity to dihedral variation, providing a basis for estimating conformer populations. All calculations were performed with the Gaussian 16 software package,^33^ except for the DLPNO–CCSD­(T)/CBS computations, which were carried out using ORCA.^34^

## 3 Results and Discussion

The *anti-gauche* conformer of 1,1,2-trifluoroethane (μ = 1.84 D), characterized by an H1–C–C–F2 dihedral angle of 54.4°, is more stable than the *double gauche* conformer (μ = 3.73 D) by 1.3 kcal mol^–1^ at the G3MP2B3 level and 1.4 kcal mol^–1^ at the B3LYP-GD3BJ/6–311++G­(d,p) level, consistent with previous reports.^18–23^ These results are also in good agreement with benchmark DLPNO–CCSD­(T)/CBS calculations, which predict a relative energy of 1.3 kcal mol^–1^. Notably, this *anti-gauche* conformer does not correspond to the structure with the highest number of C–F *gauche* interactions. As a result, unlike 1,2-difluoroethane, 1,1,2-trifluoroethane does not exhibit the classical *gauche* effect. To clarify the origin of this behavior, a detailed NBO analysis was conducted.

Although the *double gauche* conformer is more stabilized by electron delocalization than the *anti-gauche* (−187.3 vs – 183.7 kcal mol^–1^), it is also more destabilized by Lewis-type interactions (188.7 vs 183.7 kcal mol^–1^), where negative values indicate stabilization and positive values indicate destabilization. In contrast, for 1,2-difluoroethane, electron delocalization in the *gauche* conformer outweighs steric and dipolar repulsion, accounting for the classical *gauche* effect observed in this benchmark molecule. In the case of 1,1,2-trifluoroethane, however, the three interactions present in the *double gauche* conformer—compared to only one in the *anti-gauche*—are insufficient to compensate for the strong repulsive effects arising from the polar C–F bond being flanked by two vicinal C–F bonds. In the *double gauche* conformer, the three individual antiperiplanar σ_CH_ → σ*_CF_ interactions contribute 3.8–3.9 kcal mol^–1^ each, while the corresponding reciprocal σ_CF_ → σ*_CH_ interactions account for 0.6–0.7 kcal mol^–1^ each, resulting in a total stabilization energy of 13.5 kcal mol^–1^. In contrast, the *anti-gauche* conformer exhibits two σ_CH_ → σ*_CH_ interactions (1.8 and 2.2 kcal mol^–1^), two σ_CF_ → σ*_CF_ interactions (1.2 and 1.4 kcal mol^–1^), one σ_CH_ → σ*_CF_ interaction (3.6 kcal mol^–1^), and its reciprocal σ_CF_ → σ*_CH_ interaction (0.6 kcal mol^–1^), yielding a combined stabilization of 10.8 kcal mol^–1^.

Although the Lewis-type term is the primary contributor to the conformational stability of 1,1,2-trifluoroethane, the molecule’s rotational profile would be markedly different in the absence of hyperconjugation. If the electronic structure were fully localized, only a single minimum—an eclipsed conformation with an H1–C–C–F2 dihedral angle of 0°—would be observed (Figure 3). This highlights that the relative size of fluorine and hydrogen does not inherently prevent their spatial proximity, and that the electrostatic interaction between H1 (natural charge = +0.15) and F2 (−0.38) may, in fact, be attractive. In this context, electron delocalization is essential for stabilizing the staggered conformations and giving rise to the two observed energy minima.

**Figure 3:**
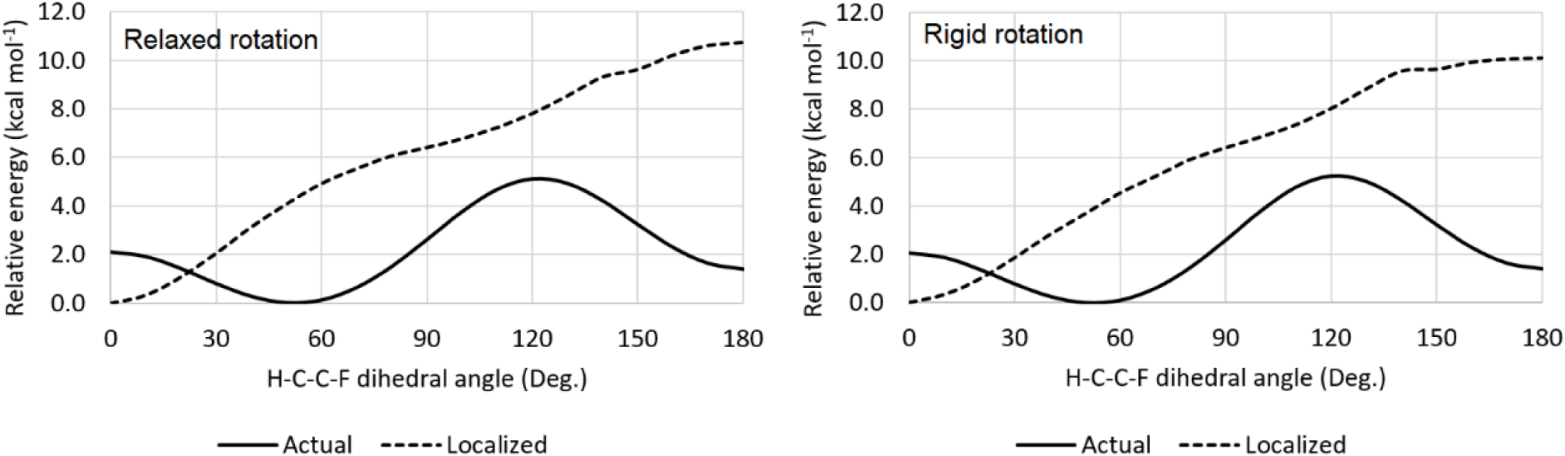
Relaxed and rigid rotational profile of 1,1,2-trifluoroethane for both the real (delocalized) and artificially localized electronic structures, computed at the B3LYP-GD3BJ/6–311++G­(d,p) level.

It is also noteworthy that C1 bears two fluorine atoms and is, therefore, more electropositive than the carbons in 1,2-difluoroethane, whose *gauche* effect has been attributed to C1···F2 electrostatic interactions.^17^ Given that these interactions are stronger in 1,1,2-trifluoroethane, and that the double-*gauche* conformer maximizes the alignment of fluorine atoms, the resulting polarization of adjacent carbon atoms is expected to further enhance conformational stability. To further verify this hypothesis, the natural charges of C1 and F2, as well as their interatomic distance, were analyzed. For the *double-gauche* conformer, q_C1_ = +0.5835 and q_F2_ = – 0.3721 (C1···F2 distance = 2.371 Å), whereas for the *anti-gauche* conformer, q_C1_ = +0.5862 and q_F2_ = −0.3756 (C1···F2 distance = 2.362 Å). According to Coulomb’s law, these values indicate a slightly stronger C1···F2 electrostatic attraction in the antigauche conformer, consistent with its higher thermodynamic stability.

It is important to note, as emphasized by Silva and coauthors,^16,35^ that properly identifying causalities in rotational profiles requires a rigid rotation around the C–C bond while keeping all other geometry parameters fixed, except for the H–C–C–F dihedral angle. Accordingly, the parameters corresponding to the eclipsed *syn* arrangement were frozen (C–C = 1.527 Å), and the C–C bond was rotated in 10° increments from *syn* to *anti* arrangements. The same procedure was applied to obtain the curve for the localized structure (Figure 3). Although the energy barriers varied — since the C–C distance particularly affects the Pauli repulsion term — the energy minima and maxima occurred at the same H–C–C–F dihedral angles as in the relaxed rotation.

Quantitative conformational analysis is typically performed using spectroscopic techniques such as infrared (IR) and nuclear magnetic resonance (NMR) spectroscopy. While population estimates based on IR spectra can be affected by differences in the molar absorptivity of the conformers,^36^ vicinal coupling constants^3^ *J*) obtained from NMR are largely insensitive to solvent effects and generally vary with conformer populations according to a Karplus relationship.^37,38^ Therefore, if an experimental^3^ *J* value is available and the individual^3^ *J* values for each conformer are calculated, the conformer populations (*n*) can be estimated using the following equations, as applied to 1,1,2-trifluoroethane:

1 JH,F(obs)3=ndouble gauche×3JH,F(double gauche)+nanti‐gauche×3JH,F(anti‐gauche)

2 $$n_{\mathrm{double gauche}}+2n_{\mathrm{anti‐gauche}}=1$$

Figure 4 shows the angular dependence of the^3^ *J*_H,F_ coupling constant in 1,1,2-trifluoroethane. Since the^3^ *J*_H,F_ values at the staggered energy minima differ significantly—approximately 13 Hz for the *double gauche* and 5 Hz for the *anti-gauche* conformer—NMR spectroscopy can reliably be employed to estimate the conformer populations at equilibrium. Because scalar spin–spin coupling constants are transmitted through the bonding framework connecting the coupled nuclei, the σ_CH_ → σ*_CF_ hyperconjugative interaction is expected to play a central role in determining the^3^ *J*_H,F_ coupling constant. Indeed, a strong correlation (*R* = 0.85) is observed between the angular dependence of^3^ *J*_H,F_ and the strength of the σ_CH_ → σ*_CF_ interaction, underscoring the influence of hyperconjugation on the rotational barrier of 1,1,2-trifluoroethane.

**Figure 4:**
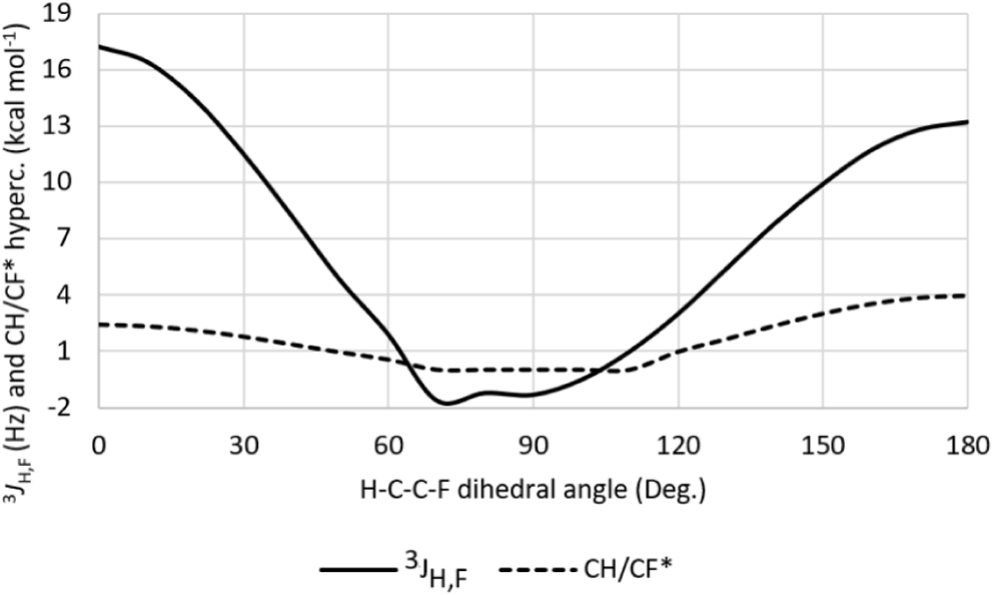
Angular dependence of the^3^ *J*_H,F_ coupling constant (Hz) and σ_CH_ → σ*_CF_ hyperconjugation (kcal mol^–1^) in 1,1,2-trifluoroethane.

Since NMR experiments are commonly conducted in chloroform (ε = 4.8) and dimethyl sulfoxide (ε = 46.7), additional SMD calculations were performed for 1,1,2-trifluoroethane to assess solvent effects (at the DFT and G3MP2B3 levels). In chloroform, the less polar medium, the *anti-gauche* conformer remains slightly favored (by 0.4 kcal mol^–1^). In contrast, in dimethyl sulfoxide the preference shifts, with the most polar *double gauche* conformer becoming more stable by 0.2–0.3 kcal mol^–1^. This reversal reflects the highly polar environment of DMSO, where dipole stabilization or attenuation of intramolecular dipole–dipole interactions in the *double gauche* conformer allows electron delocalization to outweigh the Lewis-type contribution as the dominant stabilizing factor.

The conformational behavior of 1,1,2,2-tetrafluoroethane mirrors that of 1,1,2-trifluoroethane: the conformer with fewer *gauche* C–F interactions—the *double antigauche* conformer (μ = 0.00 D)—is more stable by 1.4 kcal mol^–1^ (G3MP2B3), 1.5 kcal mol^–1^ (B3LYP-GD3BJ/6–311++G­(d,p)), or 1.4 kcal mol^–1^ (DLPNO–CCSD­(T)/CBS), again contradicting the expected fluorine *gauche* effect. In this case, the *triple gauche* conformer (μ = 2.87 D) is more strongly destabilized by Lewis-type interactions (252.0 kcal mol^–1^ vs 248.4 kcal mol^–1^ in the *double antigauche*) than it is stabilized by non-Lewis-type interactions (−250.5 kcal mol^–1^ vs – 248.4 kcal mol^–1^ in the *double antigauche*). The highly polar *triple gauche* conformer, with a dipole moment of 2.87 D, aligns all C–F bonds in roughly the same direction, thereby amplifying dipolar repulsion due to the concentration of electron density on one side of the molecule (Figure 5). This preference persists even in polar solvents, with the *double antigauche* conformer favored by 0.8 kcal mol^–1^ in implicit chloroform and by 0.3–0.4 kcal mol^–1^ in dimethyl sulfoxide.

**Figure 5:**
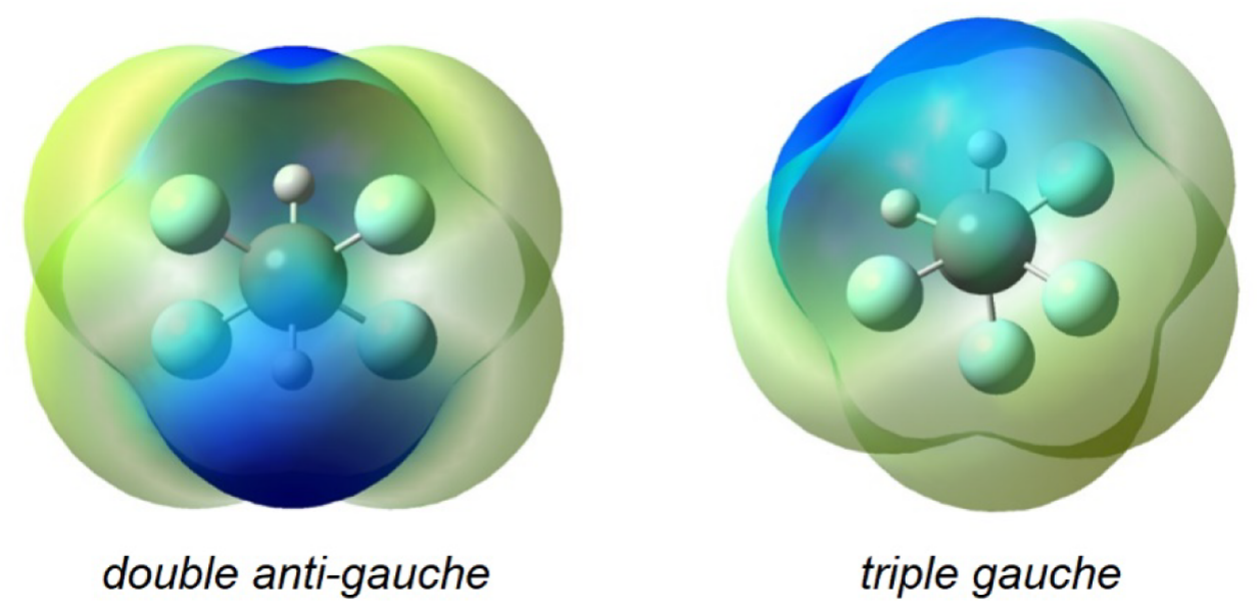
Electrostatic surface potential maps showing the charge distribution in *double antigauche* and *triple gauche* conformers of 1,1,2,2-tetrafluoroethane.

As in 1,1,2-trifluoroethane, the dominant contribution of Lewis-type interactions to the conformational stability of 1,1,2,2-tetrafluoroethane does not imply that electron delocalization is unimportant for its rotational isomerism. In fact, if the electronic structure was fully localized, only a single stable conformer—the *double antigauche*—would be expected, both in relaxed and rigid rotations with the C–C bond fixed at 1.565 Å (Figure 6). In this hypothetical scenario, an eclipsed conformer would represent the highest-energy structure due to interacting C–H and C–F bonds, while the *triple gauche* conformer would behave as a transition structure, still subject to substantial steric and dipolar repulsion. Thus, electron delocalization, which is favored in staggered geometries, plays a key role in stabilizing the *triple gauche* conformer and creating its energy minimum along the rotational profile. In the *triple gauche* conformer, two σ_CH_ → σ*_CF_ interactions contribute 2.9 kcal mol^–1^ each, complemented by two σ_CF_ → σ*_CH_ interactions of 0.6 kcal mol^–1^ and two σ_CF_ → σ*_CF_ interactions of 1.1 kcal mol^–1^, totaling 9.2 kcal mol^–1^ of stabilization. In contrast, the *double antigauche* conformer features two σ_CH_ → σ*_CH_ interactions (1.8 kcal mol^–1^ each) and four σ_CF_ → σ*_CF_ interactions (1.0 kcal mol^–1^ each), summing to 7.6 kcal mol^–1^ of stabilization.

**Figure 6:**
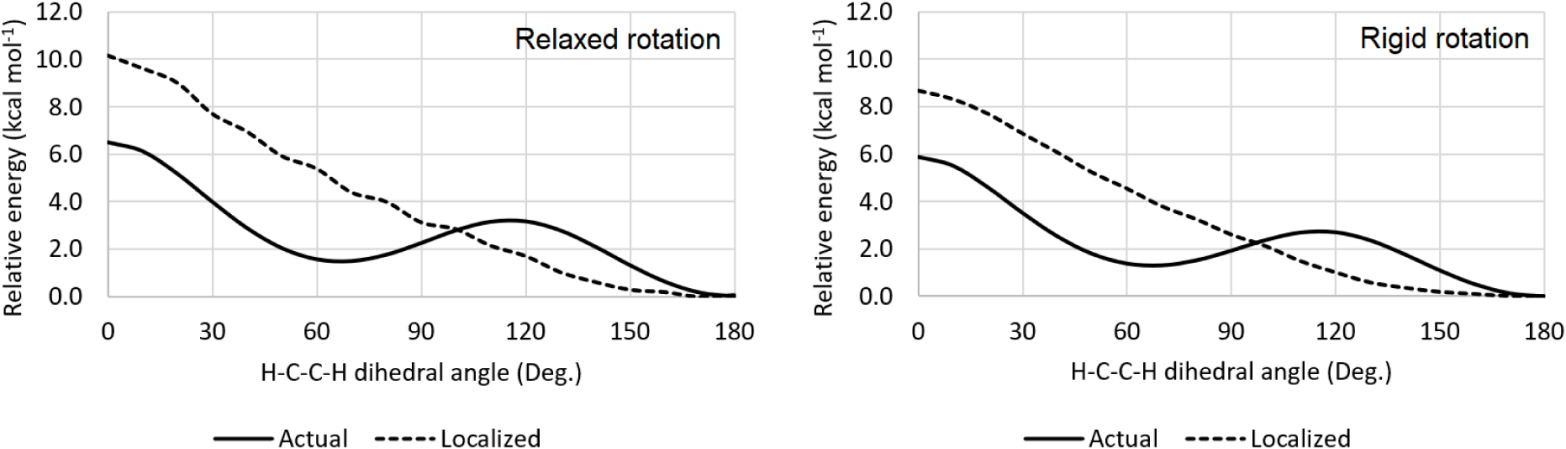
Relaxed and rigid rotational profile of 1,1,2,2-tetrafluoroethane for both the real (delocalized) and artificially localized electronic structures, computed at the B3LYP-GD3BJ/6–311++G­(d,p) level.

Similar to 1,1,2-trifluoroethane, the C1···F2 interaction in 1,1,2,2-tetrafluoroethane was analyzed as a potential source of stabilization for the double antigauche conformer relative to the triple gauche conformer. In the former, the natural charges are q_C1_ = +0.5648 and q_F2_ = – 0.3577 (C1···F2 distance = 2.343 Å), whereas in the latter, q_C1_ = +0.5651 and q_F2_(avg.) = – 0.3573 (average C1···F2 distance = 2.352 Å). These values indicate a slightly stronger C1···F2 electrostatic attraction in the double antigauche conformer, consistent with its greater conformational stability.

Given the molecular symmetry of 1,1,2,2-tetrafluoroethane, NMR coupling constants are not suitable for experimental quantitative conformational analysis. However, although not directly observable, these coupling constants can be computed and correlated with hyperconjugative interactions. Accordingly, considering the two vicinal hydrogen atoms in the molecule, the angular dependence of the^3^ *J*_H,H_ coupling constant was calculated and plotted, revealing a Karplus-like relationship (Figure 7). To examine its connection with hyperconjugation, the σ_CH_ → σ*_CH_ interaction energy was similarly plotted at 10° increments, yielding a correlation coefficient of *R* = 0.77. This correlation is slightly weaker than that found for 1,1,2-trifluoroethane, primarily because the NBO analysis imposes a threshold of 0.50 kcal mol^– 1^ for reporting individual donor–acceptor interactions; thus, any σ_CH_ → σ*_CH_ interactions below this threshold were treated as 0.0 kcal mol^– 1^, which impacts the quality of the correlation. Still, this represents a spectroscopic indication of the role of hyperconjugation in governing the conformational equilibrium of 1,1,2,2-tetrafluoroethane.

**Figure 7:**
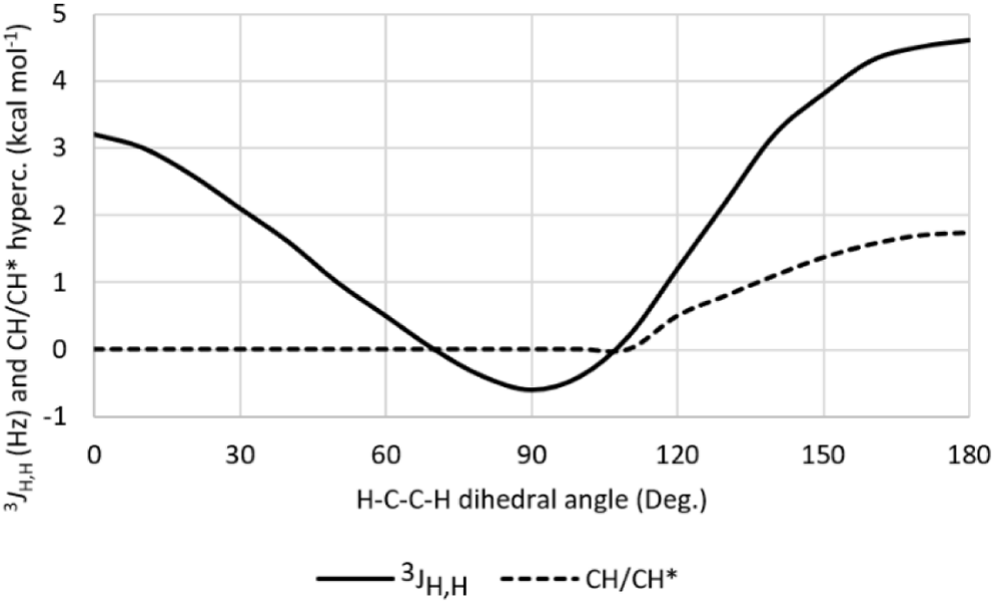
Angular dependence of the^3^ *J*_H,H_ coupling constant (Hz) and σ_CH_ → σ*_CH_ hyperconjugation (kcal mol^–1^) in 1,1,2,2-tetrafluoroethane.

## 4 Conclusions

Unlike 1,2-difluoroethane—a milestone compound renowned for its distinct conformational behavior—1,1,2-trifluoroethane and 1,1,2,2-tetrafluoroethane do not exhibit the classical *gauche* effect. This has been recognized since the term *gauche effect* was introduced in 1972, and most conformational studies on these compounds emerged during the 1990s. However, a comprehensive analysis of the steric, dipolar, and hyperconjugative contributions to their conformational preferences remained lacking, particularly since the significance of hyperconjugation in the rotational barrier of ethane was only established in 2001.

In this study, the conformational preferences of 1,1,2-trifluoroethane and 1,1,2,2-tetrafluoroethane were analyzed through the lens of Natural Bond Orbital (NBO) theory. Lewis-type interactions—especially dipolar repulsion—emerged as the dominant factor governing the *anti-gauche* preference in the trifluorinated compound and the *double antigauche* preference in the tetra-fluorinated analogue. Nevertheless, in the absence of electron delocalization, only a single conformer would be expected for each molecule—an eclipsed form for 1,1,2-trifluoroethane and the *double antigauche* form for 1,1,2,2-tetrafluoroethane. This highlights that while electron delocalization is not the principal stabilizing force, it is essential for the existence and energy minimization of staggered conformers. Calculated vicinal NMR coupling constants support these conclusions, reinforcing the role of hyperconjugation in shaping the rotational landscape of these fluorinated ethanes.

## Supplementary Material



## Notes

Standard coordinates and absolute energies of the optimized geometries (PDF)
