# Supplementary material for: Revisiting the Conformational Equilibrium of 1,1,2-Trifluoroethane and 1,1,2,2-Tetrafluoroethane: An NBO Study
Source: J Phys Chem A. 2025 Oct 23;129(44):10181–6. doi: 10.1021/acs.jpca.5c06020 (PMC12598850; doi:10.1021/acs.jpca.5c06020)
Supplement: Supplementary file 1 [file jp5c06020_si_001.pdf]

SUPPORTING INFORMATION

Revisiting the Conformational Equilibrium of 1,1,2-Trifluoroethane and 1,1,2,2-Tetrafluoroethane: An NBO Study

Matheus P. Freitas[a]*

[a] Department of Chemistry, Institute of Natural Sciences, Federal University of Lavras, 37200-900, Lavras, MG, Brazil

* E-mail: matheus@ufla.br

Pages S2–S13. Standard Cartesian coordinates of the optimized geometries for the conformers of 1,1,2-trifluoroethane and 1,1,2,2-tetrafluoroethane, along with their respective standard Gibbs free energies.

1,1,2-Trifluoroethane *double gauche* (G3MP2B3)

0 1

C -0.74290400 -0.83154600 0.00000000

H -1.33834200 -1.05018000 -0.89499000

H -1.33834200 -1.05018000 0.89499000

C -0.35757700 0.63322800 0.00000000

H -1.24396400 1.28175100 0.00000000

F 0.38976000 0.92001800 -1.10395100

F 0.38976000 0.92001800 1.10395100

F 0.38976000 -1.61686600 0.00000000

G^0 = -377.173654 hartrees

1,1,2-Trifluoroethane *anti gauche* (G3MP2B3)

0 1

C -0.77022600 -0.60106300 -0.27527000

H -0.85487000 -1.64541600 0.04664300

H -0.69570800 -0.55674200 -1.36816100

C 0.47121700 0.02174200 0.33109000

H 0.42010100 0.10190500 1.42297900

F 0.66254700 1.26915600 -0.18233400

F 1.54420000 -0.75388400 -0.01180100

F -1.88179800 0.10430400 0.14564800

G^0 = -377.175711 hartrees

1,1,2-Trifluoroethane *double gauche* (B3LYP-GD3BJ/6-311++G(d,p))

0 1

C 0.73888300 -0.83684500 0.00000000

H 1.32550600 -1.05615300 0.89576800

H 1.32550600 -1.05615300 -0.89576800

C 0.35925000 0.62870400 0.00000000

H 1.24625000 1.26982000 0.00000000

F -0.38837200 0.93522700 1.10241400

F -0.38837200 0.93522700 -1.10241400

F -0.38837200 -1.63808400 0.00000000

G^0 = -377.639530 hartrees

1,1,2-Trifluoroethane *anti gauche* (B3LYP-GD3BJ/6-311++G(d,p))

0 1

C -0.77694100 -0.58505600 -0.29318700

H -0.86849700 -1.62915700 0.01259000

H -0.72393200 -0.50637500 -1.38097600

C 0.46482100 0.01997200 0.32858300

H 0.41388500 0.10345300 1.41595700

F 0.69734600 1.26482000 -0.18206600

F 1.53510000 -0.77233800 -0.00340100

F -1.89341800 0.11002700 0.15658400

G^0 = -377.641807 hartrees

1,1,2,2-Trifluoroethane *double anti-gauche* (G3MP2B3)

0 1

C 0.24454000 -0.72172200 0.00000000

H 1.33827500 -0.77928900 0.00000000

C -0.24454000 0.72172200 0.00000000

H -1.33827500 0.77928900 0.00000000

F 0.24454000 1.34397000 -1.10550500

F 0.24454000 1.34397000 1.10550500

F -0.24454000 -1.34397000 1.10550500

F -0.24454000 -1.34397000 -1.10550500

G^0 = -476.347652 hartrees

1,1,2,2-Trifluoroethane *triple gauche* (G3MP2B3)

0 1

C -0.20309200 0.73599900 -0.35308200

H 0.23393200 1.27205000 -1.20269800

C 0.20309200 -0.73599900 -0.35308200

H -0.23393200 -1.27205000 -1.20269800

F -0.20309200 -1.32136800 0.79988000

F 1.56160800 -0.80658200 -0.43085900

F -1.56160800 0.80658200 -0.43085900

F 0.20309200 1.32136800 0.79988000

G^0 = -476.345375 hartrees

1,1,2,2-Trifluoroethane *double anti-gauche* (B3LYP-GD3BJ/6-311++G(d,p))

0 1

C 0.24639300 0.72355000 0.00000000

H 1.33615400 0.79262700 0.00000000

C -0.24639300 -0.72355000 0.00000000

H -1.33615400 -0.79262700 0.00000000

F 0.24639300 -1.35234000 1.10221100

F 0.24639300 -1.35234000 -1.10221100

F -0.24639300 1.35234000 -1.10221100

F -0.24639300 1.35234000 1.10221100

G^0 = -476.922716 hartrees

1,1,2,2-Trifluoroethane *triple gauche* (B3LYP-GD3BJ/6-311++G(d,p))

0 1

C -0.19936900 0.73896600 -0.35049100

H 0.23856500 1.26989700 -1.19908900

C 0.19936900 -0.73896600 -0.35049100

H -0.23856500 -1.26989700 -1.19908900

F -0.19936900 -1.33767700 0.79839900

F 1.55923800 -0.82007400 -0.43150700

F -1.55923800 0.82007400 -0.43150700

F 0.19936900 1.33767700 0.79839900

G^0 = -476.920284 hartrees

1,1,2-Trifluoroethane *double gauche* (G3MP2B3) – chloroform (SMD)

0 1

C 0.75321300 -0.82784200 0.00000000

H 1.34285400 -1.05213700 0.89522400

H 1.34285400 -1.05213700 -0.89522400

C 0.36700800 0.63170600 0.00000000

H 1.24408100 1.28732900 0.00000000

F -0.39448600 0.91581300 1.10318100

F -0.39448600 0.91581300 -1.10318100

F -0.39448600 -1.61009700 0.00000000

G^0 = -377.182190 hartrees

1,1,2-Trifluoroethane *anti gauche* (G3MP2B3) – chloroform (SMD)

0 1

C -0.76342500 -0.62014600 -0.26518500

H -0.85624900 -1.65241900 0.08591400

H -0.69036400 -0.60070400 -1.35749400

C 0.46885400 0.01827800 0.33614400

H 0.42996100 0.09939600 1.42593400

F 0.64292300 1.27308400 -0.18275800

F 1.55599900 -0.73841600 -0.01874800

F -1.87846900 0.10588000 0.13704800

G^0 = -377.182757 hartrees

1,1,2-Trifluoroethane *double gauche* (B3LYP-GD3BJ/6-311++G(d,p)) – chloroform (SMD)

0 1

C 0.75164700 -0.83194500 0.00000000

H 1.33161600 -1.05841100 0.89672300

H 1.33161600 -1.05841100 -0.89672300

C 0.37136600 0.62626100 0.00000000

H 1.24894100 1.27582200 0.00000000

F -0.39445400 0.93085000 1.10158100

F -0.39445400 0.93085000 -1.10158100

F -0.39445400 -1.63113200 0.00000000

G^0 = -377.649269 hartrees

1,1,2-Trifluoroethane *anti gauche* (B3LYP-GD3BJ/6-311++G(d,p)) – chloroform (SMD)

0 1

C -0.76877600 -0.60858700 -0.27978300

H -0.87157100 -1.63932900 0.06212600

H -0.71617900 -0.56150300 -1.36876900

C 0.46178100 0.01464500 0.33652100

H 0.42639700 0.10143600 1.42259500

F 0.67472100 1.26911100 -0.18335500

F 1.54970000 -0.75304700 -0.01243700

F -1.89071900 0.11316400 0.14508200

G^0 = -377.649916 hartrees

1,1,2,2-Tetrafluoroethane *double anti-gauche* (G3MP2B3) – chloroform (SMD)

0 1

C 0.24740500 0.71974900 0.00000000

H 1.33893100 0.78340300 0.00000000

C -0.24740500 -0.71974900 0.00000000

H -1.33893100 -0.78340300 0.00000000

F 0.24740500 -1.34522900 1.10604700

F 0.24740500 -1.34522900 -1.10604700

F -0.24740500 1.34522900 -1.10604700

F -0.24740500 1.34522900 1.10604700

G^0 = -476.353637 hartrees

1,1,2,2-Tetrafluoroethane *triple gauche* (G3MP2B3) – chloroform (SMD)

0 1

C -0.21280800 0.73141200 -0.36902000

H 0.18941300 1.27648700 -1.22728500

C 0.21280800 -0.73141200 -0.36902000

H -0.18941300 -1.27648700 -1.22728500

F -0.21280800 -1.32415900 0.78090100

F 1.57691200 -0.78099500 -0.39852200

F -1.57691200 0.78099500 -0.39852200

F 0.21280800 1.32415900 0.78090100

G^0 = -476.352447 hartrees

1,1,2,2-Tetrafluoroethane *double anti-gauche* (B3LYP-GD3BJ/6-311++G(d,p)) – chloroform (SMD)

0 1

C 0.25000500 0.72109000 0.00000000

H 1.33787700 0.79732500 0.00000000

C -0.25000500 -0.72109000 0.00000000

H -1.33787700 -0.79732500 0.00000000

F 0.25000500 -1.35401500 1.10320100

F 0.25000500 -1.35401500 -1.10320100

F -0.25000500 1.35401500 -1.10320100

F -0.25000500 1.35401500 1.10320100

G^0 = -476.929765 hartrees

1,1,2,2-Tetrafluoroethane *triple gauche* (B3LYP-GD3BJ/6-311++G(d,p)) – chloroform (SMD)

0 1

C -0.20806300 0.73457300 -0.36799700

H 0.19556600 1.27453500 -1.22520400

C 0.20806300 -0.73457300 -0.36799700

H -0.19556600 -1.27453500 -1.22520400

F -0.20806300 -1.34023800 0.78067000

F 1.57507300 -0.79622400 -0.39920500

F -1.57507300 0.79622400 -0.39920500

F 0.20806300 1.34023800 0.78067000

G^0 = -476.928435 hartrees

1,1,2-Trifluoroethane *double gauche* (G3MP2B3) – DMSO (SMD)

0 1

C 0.75776100 -0.82588200 0.00000000

H 1.34520000 -1.05224100 0.89522700

H 1.34520000 -1.05224100 -0.89522700

C 0.37165900 0.63127400 0.00000000

H 1.24455700 1.28997200 0.00000000

F -0.39672100 0.91315300 1.10253300

F -0.39672100 0.91315300 -1.10253300

F -0.39672100 -1.60606500 0.00000000

G^0 = -377.183495 hartrees

1,1,2-Trifluoroethane *anti gauche* (G3MP2B3) – DMSO (SMD)

0 1

C -0.75998400 -0.62846800 -0.25985800

H -0.85706900 -1.65562300 0.10302200

H -0.68629100 -0.61947700 -1.35197600

C 0.46833400 0.01613100 0.33925300

H 0.43530800 0.09808900 1.42854200

F 0.63282000 1.27374800 -0.18281700

F 1.56114500 -0.72998000 -0.02268800

F -1.87641400 0.10634600 0.13262000

G^0 = -377.183137 hartrees

1,1,2-Trifluoroethane *double gauche* (B3LYP-GD3BJ/6-311++G(d,p)) – DMSO (SMD)

0 1

C 0.75768800 -0.82953700 0.00000000

H 1.33494200 -1.05850400 0.89686300

H 1.33494200 -1.05850400 -0.89686300

C 0.37744900 0.62569100 0.00000000

H 1.25010700 1.27930300 0.00000000

F -0.39743800 0.92775900 1.10086700

F -0.39743800 0.92775900 -1.10086700

F -0.39743800 -1.62654100 0.00000000

G^0 = -377.651120 hartrees

1,1,2-Trifluoroethane *anti gauche* (B3LYP-GD3BJ/6-311++G(d,p)) – DMSO (SMD)

0 1

C -0.76495300 -0.61966000 -0.27251700

H -0.87226600 -1.64352400 0.08619200

H -0.71139700 -0.58781800 -1.36167200

C 0.46114000 0.01185800 0.34014700

H 0.43259900 0.09941400 1.42555900

F 0.66288500 1.27123600 -0.18334500

F 1.55644300 -0.74340200 -0.01746000

F -1.88889000 0.11424800 0.13904200

G^0 = -377.650698 hartrees

1,1,2,2-Tetrafluoroethane *double anti-gauche* (G3MP2B3) – DMSO (SMD)

0 1

C 0.24834400 0.71910100 0.00000000

H 1.33908100 0.78437600 0.00000000

C -0.24834400 -0.71910100 0.00000000

H -1.33908100 -0.78437600 0.00000000

F 0.24834400 -1.34583200 1.10620400

F 0.24834400 -1.34583200 -1.10620400

F -0.24834400 1.34583200 -1.10620400

F -0.24834400 1.34583200 1.10620400

G^0 = -476.353867 hartrees

1,1,2,2-Tetrafluoroethane *triple gauche* (G3MP2B3) – DMSO (SMD)

0 1

C 0.21476800 0.73007900 -0.37719800

H 0.83444700 0.97978400 -1.24142800

C -0.21476800 -0.73007900 -0.37719800

H -0.83444700 -0.97978400 -1.24142800

F -0.90826800 -0.99201800 0.76913600

F 0.90826800 -1.50751000 -0.37973400

F -0.90826800 1.50751000 -0.37973400

F 0.90826800 0.99201800 0.76913600

G^0 = -476.353426 hartrees

1,1,2,2-Tetrafluoroethane *double anti-gauche* (B3LYP-GD3BJ/6-311++G(d,p)) – DMSO (SMD)

0 1

C 0.25122700 0.72041000 0.00000000

H 1.33834500 0.79843400 0.00000000

C -0.25122700 -0.72041000 0.00000000

H -1.33834500 -0.79843400 0.00000000

F 0.25122700 -1.35494900 1.10348400

F 0.25122700 -1.35494900 -1.10348400

F -0.25122700 1.35494900 -1.10348400

F -0.25122700 1.35494900 1.10348400

G^0 = -476.930397 hartrees

1,1,2,2-Tetrafluoroethane *triple gauche* (B3LYP-GD3BJ/6-311++G(d,p)) – DMSO (SMD)

0 1

C -0.21456100 0.73183000 -0.37773300

H 0.16934900 1.27522000 -1.24059000

C 0.21456100 -0.73183000 -0.37773300

H -0.16934900 -1.27522000 -1.24059000

F -0.21456100 -1.34267100 0.76886600

F 1.58293600 -0.78178600 -0.37920100

F -1.58293600 0.78178600 -0.37920100

F 0.21456100 1.34267100 0.76886600

G^0 = -476.929766 hartrees
